# Roles of Prohibitin in Growth Control and Tumor Suppression in Human Cancers

**Published:** 2008-02-10

**Authors:** Sheng Wang, Douglas V. Faller

**Affiliations:** Boston University School of Medicine, Cancer Research Center, Boston, MA, U.S.A

## Abstract

Tumor formation results from alterations in the normal control of cell proliferation. In the past decade, much attention in cancer research has been focused on the function of proto-oncogenes and tumor suppressors. Prohibitin is a potential tumor suppressor which was originally identified because of its anti-proliferative activities. Subsequent investigations led to the discovery of prohibitin mutations in sporadic breast cancers. Recent studies established that prohibitin directly regulates E2F-mediated transcription and growth suppression Prohibitin further attracted the attention of the translational cancer research community when it was recently connected to the regulation of estrogen receptor and androgen receptor activity. Prohibitin was shown to be required for the growth suppression of breast cancer cells induced by estrogen antagonists, and for therapeutic responses to androgen antagonists in prostate cancer. Through the application of new molecular technologies, additional novel functions of prohibitin have been revealed, demonstrating diverse and essential roles of this highly-conserved protein in regulating cell growth.

## Introduction

The human prohibitin gene was identified and cloned in 1991, as the result of a search for potential tumor suppressors, on the basis of its anti-proliferative activities (Nuell et al. 1991). Later studies revealed that prohibitin represses cell growth by modulating E2F transcriptional activity (Wang et al. 1999a; Wang et al. 1999b). The molecular mechanisms of the prohibitin-mediated transcriptional repression and growth suppression have now been extensively characterized, revealing that prohibitin recruits chromatin-remodeling molecules to gene promoter elements for transcriptional repression (Wang et al. 2002a; Wang et al. 2002b).

Prohibitin was first linked to human cancers by the discoveries of prohibitin mutations in breast cancers (Sato et al. 1992; Sato et al. 1993). Later studies indicated that prohibitin and its co-repressors are required for the growth suppression induced by estrogen antagonists (Wang et al. 2004). Recent investigations have demonstrated that prohibitin associates with the estrogen receptor-α (ERα) and participates in ER-mediated transcriptional regulation (Zhang et al. 2007). Prohibitin has also been linked to human prostate cancer through recent investigations establishing that prohibitin associates with the androgen receptor (AR) and participates in AR-mediated gene regulation (Gamble et al. 2007; Gamble et al. 2004).

In addition to transcriptional repression, prohibitin can induce p53-mediated transcription, indicating that prohibitin may have dual functions in modulating transcription (Fusaro et al. 2003; Fusaro et al. 2002). This theory is further supported by the recent demonstration that prohibitin differentially regulates the Yin-Yang1 (YY1) and caspase 7 gene promoter activities (Joshi et al. 2007).

Additional diverse functions of prohibitin were recently reported, which link this evolutionally highly-conserved gene to apoptosis (Chowdhury et al. 2007; Fusaro et al. 2002), to signal transduction through the MAPK pathway (Rajalingam and Rudel, 2005; Rajalingam et al. 2005; Wang et al. 1998; Wang et al. 1999b), and to mitochondrial biogenesis (Ahn et al. 2006).

The critical functions of prohibitin in transcriptional regulation and growth control indicate the importance of prohibitin-directed research and translational investigation to further clarify the role of prohibitin in cancer development.

## Molecular Biology of Prohibitin

In an attempt to identify potential tumor suppressors, the rat prohibitin gene was first cloned based on its anti-proliferative activity when overexpressed (McClung et al. 1992; Nuell et al. 1991). Microinjection of prohibitin mRNA into cell nuclei blocked S phase entry, while down-regulation of prohibitin *via* anti-sense stimulated cell entry into S phase (Nuell et al. 1991). The human prohibitin gene was identified soon afterwards and cloned based on its homology to the rat prohibitin gene (Sato et al. 1992; Sato et al. 1993). The prohibitin gene is highly-conserved from yeast to human, and is an analog of Cc, a Drosophila melanogaster gene required for normal development (Eveleth and Marsh, 1986).

The molecular mechanisms underlying prohibitin-mediated growth suppression have been extensively studied over the past few years, revealing that prohibitin collaborates with chromatin remodeling molecules and regulates transcription (Wang et al. 2002a; Wang et al. 1999a; Wang et al. 1999b; Wang et al. 1999c; Wang et al. 2002b; Wang et al. 2004; Zhang et al. 2007).

**Figure 1:**
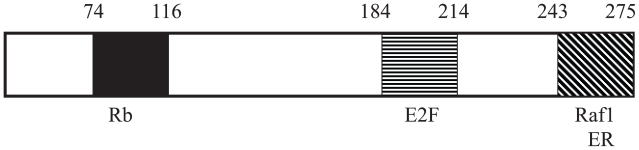
Regions of prohibitin involved in binding to E2F, ER, Raf1, and Rb proteins Regions of the prohibitin protein responsible for binding to the Rb tumor suppressor, the E2F transcription factors, the Raf1 protein, and the estrogen receptor (ER) are indicated.

Associations between prohibitin and many other proteins have been established (Fusaro et al. 2003; Fusaro et al. 2002; Gamble et al. 2007; Gamble et al. 2004; Joshi et al. 2007; Rasmussen et al. 1998; Rastogi et al. 2006a; Rastogi et al. 2006b; Wang et al. 2002a; Wang et al. 1999a; Wang et al. 1999b; Wang et al. 1999c; Wang et al. 2002b; Wang et al. 2004; Zhang et al. 2007). These prohibitin-associated proteins include established critical factors in transcription regulation, growth control, apoptosis and signal transductions (Table 1 and Fig. 1).

### Prohibitin and E2F/Rb

**Figure 2:**
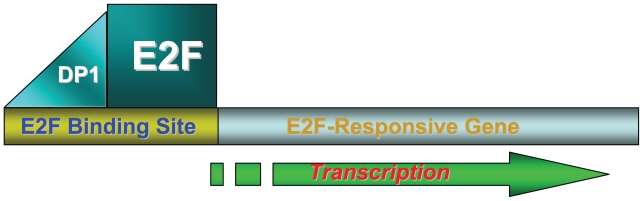
E2F-Dependent Transcriptional Activation E2F binds to E2F-binding sites in the promoter regions of E2F-responsive genes, in cooperation with the DP1 dimerization partner protein, to elicit transcriptional activation.

The anti-proliferative activities and evolutionary conservation of prohibitin attracted the attention of researchers in the field of cell cycle control and cancer development, resulting in the discovery of the relationship between prohibitin and the E2F pathway (Wang et al. 1999a; Wang et al. 1999b). The fundamental importance of E2F family of transcriptional factors in cell cycle control, apoptosis, differentiation and transformation has been well established (Berk, 2005; Dannenberg and te Riele, 2006; Du and Pogoriler, 2006; Gladden and Diehl, 2005; Halaban, 2005; Harbour and Dean, 2000; Johnson and Schneider-Broussard, 1998; Khidr and Chen, 2006; Korenjak and Brehm, 2005; Macleod, 1999; Muller and Helin, 2000; Nevins, 2001; Nevins et al. 1997; Qin et al. 1995; Rogoff and Kowalik, 2004; Ruiz et al. 2006; Sebastian and Johnson, 2006; Sellers and Kaelin, 1997; Wang et al. 1999c; Zhu, 2005). E2F activity is essential for the expression of critical cellular genes required for progression into, and through, the DNA-synthetic S-phase of cell cycle. The Rb family of tumor suppressors interacts with the E2F family members and regulates their function in order to control cell cycle progression (Fig. 2) (Wang et al. 1999a; Wang et al. 1999b).

In an attempt to identify factors that associate with Rb family of tumor suppressors, using the yeast two-hybrid system, we and others initially discovered that prohibitin associates with p130, a member of the Rb family (Wang et al. 1999a; Wang et al. 1999b). Later studies established that prohibitin associates with all three members of Rb family, both *in vitro* and *in vivo*. We therefore investigated whether prohibitin participates in the regulation of E2F, and demonstrated that prohibitin specifically represses E2F-mediated transcription and growth (Wang et al. 1999a; Wang et al. 1999b).

A number of findings now mechanistically distinguish the regulation of E2F transcriptional activity by prohibitin, in comparison to the regulation of E2F by Rb family members:

- Prohibitin and Rb target different regions of the E2F molecule; Rb interacts with and, targets, the trans-activation domain of E2F, at its C-terminus, while prohibitin targets the highly-conserved “Marked-box” domain, in the center of the E2F protein (Wang et al. 1999b).
- Rb is regulated by cyclins and cyclin-dependent kinases (Yamasaki, 2003). In contrast, prohibitin responds to a different set of signals, and not to cyclin-dependent kinases (Wang et al. 1999b).
- The finding that prohibitin associates with the IgM receptor suggested that prohibitin may play a role in IgM signaling (McClung et al. 1995; Terashima et al. 1994). Later studies indicated that IgM stimulation can reverse prohibitin-mediated, but not Rb-mediated, E2F repression (Wang et al. 1999b).
- Rb is targeted by viral onco-proteins such as E1A, SV40Tag, and E7 (Berk, 2005; Dannenberg and te Riele, 2006; Dasgupta et al. 2006; Dasgupta et al. 2004; Delston and Harbour, 2006; Du and Pogoriler, 2006; Galderisi et al. 2006; Halaban, 2005; Khidr and Chen, 2006; Korenjak and Brehm, 2005; Korenjak and Brehm, 2006; Sebastian and Johnson, 2006; Yamasaki, 2003; Zhu, 2005). In contrast, prohibitin-mediated repression of E2F transcriptional activity is not affected by E1A, suggesting that prohibitin represses E2F activity through mechanisms distinct from those utilized by the Rb family members (Wang et al. 1999a).
- The MAPK pathway is intimately connected to cell cycle progression, and we and others recently established that Raf1, a central molecule of the MAPK pathway, associates with Rb and regulates its activity by phosphorylation (Wang et al. 1998). Studies of potential roles of Raf1 in the prohibitin-mediated E2F modulation revealed that Raf1 also associates with prohibitin and reverses its repressive effects on E2F-mediated gene transcription and growth (Wang et al. 1998). Interestingly, further investigation revealed that different regions of Raf1 are involved in its binding to Rb compared to prohibitin. Raf1 targets the C-terminus of prohibitin, while Rb associates with prohibitin *via* a domain located in the middle of the protein (Fig. 1) (Wang et al. 1999b). These finding suggest the ability of prohibitin-mediated E2F transcriptional repression to be responsive to a different set of signal transduction events than is the repression conferred by the Rb family members. The molecular mechanisms of Raf-mediated prohibitin regulation are not yet fully elucidated. Recent studies find that prohibitin is required for Raf1 activation by Ras signaling, further demonstrating the inter-play between prohibitin and the MAPK pathway (further discussed in Section V).

**Figure 3:**
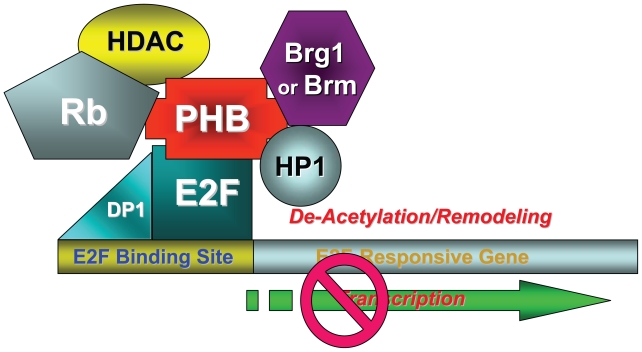
Repression of E2F-Dependent Transcription by Prohibitin Prohibitin recruits chromatin remodeling molecules for the transcriptional repression of E2F-responsive genes. **Abbreviations:** PHB: prohibitin; Rb: retinoblastoma protein; HP1: Heterochromatin protein 1; HDAC: Histone deacetylase.

The molecular mechanism of prohibitin-mediated suppression of E2F-mediated gene transcription and cellular growth have been more fully elucidated through our utilization of the recently-developed chromatin immuno-precipitation (ChIP) assay, which reveals that prohibitin recruits chromatin remodeling molecules (HDAC1, Brg1, Brm, and HP1) to E2F-responsive promoters. In addition, the use of siRNA techniques for selective knock-out of proteins has allowed us to demonstrate that prohibitin requires association with chromatin-remodeling molecules for its effects on transcriptional regulation and growth suppression (Fig. 3) (Wang et al. 2002a; Wang et al. 2002b; Wang et al. 2004; Zhang et al. 2007).

### Prohibitin and p53

The essential role of p53 in the development of apoptosis is well established, and cooperation between E2F1 and p53 has been demonstrated (Berk, 2005; Funayama and Ishikawa, 2007; Harris and Levine, 2005; Rogoff and Kowalik, 2004; Stanelle and Putzer, 2006; Sugimoto et al. 2006), raising the possibility that prohibitin, as an E2F repressor, might play a role in E2F1/p53-mediated apoptosis. Studies addressing this question revealed that prohibitin associates with p53 and enhances p53 transcriptional activities by increasing the ability of p53 to bind to its consensus sites on DNA (Fusaro et al. 2003; Fusaro et al. 2002; Joshi et al. 2007). This discovery was the first indication that prohibitin can also function as a transcription activator, in addition to being a transcriptional repressor, which places prohibitin in a unique position to mediate cross-talk between the E2F and p53 pathways. Similar opposing, promoter-specific regulatory activities of prohibitin have been further demonstrated in a recent report that prohibitin represses Yin-Yang1 gene promoter activity *via* newly-identified E2F-binding sites, while enhancing levels of caspase 7 promoter activity through p53, *via* p53-binding sites (Joshi et al. 2007).

Previous studies indicated that prohibitin may associate with the inner membrane of mitochondria, a node where pro-apoptotic signals converge, under certain circumstances. Sub-cellular localization studies of prohibitin revealed that the protein is predominantly nuclear, where it co-localizes with E2F1 and p53. In response to apoptotic stimuli, however, prohibitin is exported from the nucleus and localizes to the mitochondria, indicating a potential connection between the differential localization of prohibitin and its roles in both growth suppression and apoptosis (Joshi et al. 2007). Further investigation to establish a causal relationship between sub-cellular localization of prohibitin and cellular fate may lead to discovery of novel regulatory machinery bridging the E2F and p53 pathways in the processes of cell cycle modulation and apoptosis.

## Prohibitin and Breast Cancer

Previous genetic studies revealed that the prohibitin gene is located at chromosomal position 17q21-q22, a region genetically linked to early-onset human breast cancer (Sato et al. 1992; White et al. 1991). Investigation of a possible connection between prohibitin and breast cancer revealed mutations of prohibitin genes in primary breast cancers, indicating that prohibitin may play important roles in breast cancer development (Sato et al. 1992; Sato et al. 1993). Our studies later revealed that prohibitin is required for the growth suppression of breast cancer cells induced by estrogen antagonists (Wang et al. 2004). More recently, we have shown that prohibitin participates in estrogen receptor (ER)-mediated transcription regulation (Zhang et al. 2007).

### Mutations of prohibitin in breast cancers

**Figure 4:**
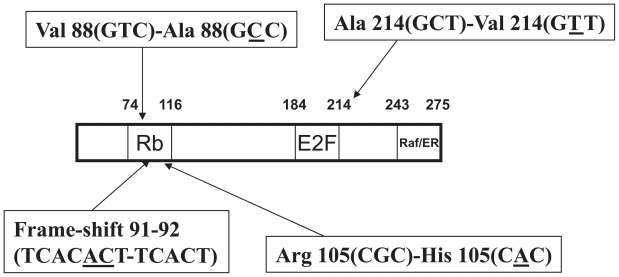
Somatic mutations of prohibitin identified in breast cancers Four reported mutations of prohibitin identified in breast tumors are illustrated.

To investigate potential clinical importance of prohibitin in breast cancer development, tumors from twenty-three patients with sporadic breast cancer were surveyed and four somatic mutations of the prohibitin gene were identified (Sato et al. 1992; Sato et al. 1993). Among the mutations detected, two were mis-sense mutations, in one case a 2-base deletion resulting in truncation of the gene product due to a frame shift; and in the second, a C to T transition in an intron adjacent to an intron-exon boundary. One additional point mutation (Ala-Val) was identified by the same group in a subsequent larger scale survey (Sato et al. 1992; Sato et al. 1993). The effects of these mutations on prohibitin function remain currently unknown. Interestingly, three of the four exon mutations were located in the Rb-binding domain and the last was within the E2F-binding region (Fig. 4). These results suggest that prohibitin may be a tumor suppressor, associated with breast cancer development and/or progression. Further large-scale translational-clinical investigation and analysis of the functional significance of the mutations identified to date are required for a better understanding of the relationship between mutations in prohibitin and breast cancers.

### Roles of prohibitin in estrogen antagonist-mediated growth suppression

Estrogen antagonists, such as tamoxifen, are the most commonly prescribed agents for the treatment and prevention of breast cancer. Although most patients with tumors expressing the estrogen receptor initially respond to estrogen antagonists, the inevitable development of resistance limits their long-term use. The molecular mechanisms of tamoxifen-mediated tumor growth suppression have not been fully elucidated (Ali and Coombes, 2002; Ariazi et al. 2006; Boccardo et al. 2005; Cuzick et al. 2006; Ellis, 2004; Gradishar, 2004; Howell et al. 2005; Hussain et al. 2004; Kurebayashi, 2003; Lofgren et al. 2006; Mousavi and Rezaei, 2006; O’Regan, 2006; Osborne et al. 2005; Robertson et al. 2005; Saeki et al. 2005; Schiff et al. 2003; Wang et al. 2004). Because mutations of prohibitin have been linked to breast cancer development, the potential involvement of prohibitin in estrogen antagonist-induced growth suppression was explored.

Previous studies indicated that estrogen antagonists induced growth suppression at G1, which coincides with the phase of cell cycle arrest induced during prohibitin-mediated repression of cell cycle progression (Saeki et al. 2005; Wang et al. 2004), suggesting that the two processes might be functionally related. We found that depletion of prohibitin from tamoxifen-sensitive breast cancer cells using siRNA prevented tamoxifen-induced cell cycle arrest, demonstrating a necessary role for prohibitin in estrogen antagonist-induced growth suppression (Wang et al. 2004). E2F is a critical regulator of the G1-S transition, and as discussed above, prohibitin is a well-established repressor of E2F activity. Studies were therefore undertaken to determine whether estrogen antagonists target the E2F pathway, and confirmed that E2F transcriptional activity is indeed repressed by exposure to estrogen antagonists, and that this repression requires prohibitin (Wang et al. 2004).

Prohibitin recruits Brg1 and Brm to E2F-responsive promoters for repression of E2F transcriptional activity (Wang et al. 2002b). Brg1 and Brm are the core ATPase components of the SWI/ SNF complex family, and mediate transcriptional regulation *via* local chromatin remodeling (Dasgupta et al. 2004; Dunaief et al. 1994; Giacinti and Giordano, 2006; Inayoshi et al. 2006; Wang et al. 2004; Zhang et al. 2007). Earlier studies indicated that Brg1 and Brm were primarily involved in the activation of transcription, while more recent results demonstrate that they also function as co-repressors with prohibitin and Rb in the regulation of E2F activity (Wang et al. 2004). Mutations or silencing of Brg1, Brm and other members of SWI/SNF have been found in many types of cancers, including breast cancers (Inayoshi et al. 2006). To test the potential involvement of Brg1 and Brm in estrogen antagonist-induced E2F repression and growth suppression, we employed siRNA and dominant-negative mutation strategies to suppress endogenous Brg1 and/ or Brm levels, and demonstrated that both of these two chromatin remodeling molecules are required for estrogen antagonist-induced E2F transcriptional repression and growth suppression. Treatment of breast cancer cells with estrogen antagonists induced a temporal association between prohibitin and its co-repressors (Brg1, Brm), which correlates with the recruitment of Brg1 and Brm to endogenous E2F-responsive promoters. These findings demonstrate the essential importance of the prohibitin/E2F pathway in estrogen antagonist-induced growth suppression, and identify prohibitin and Brg1/Brm as potential novel targets for the design of improved anti-breast cancer therapy (Wang et al. 2004). However the clinical importance of the prohibi-tin/Brg1/Brm and E2F axis in the treatment of breast cancer remains to be examined.

### Regulation of estrogen receptor (ER) activity by prohibitin

**Figure 5:**
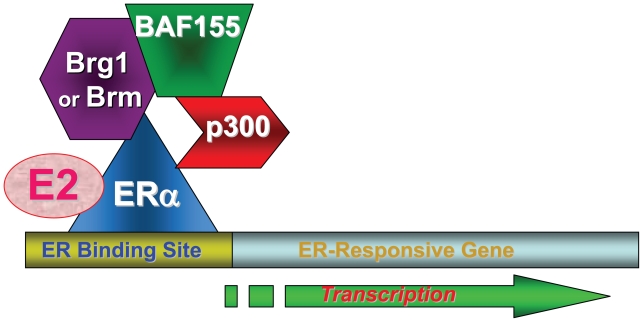
ER-Dependent Transcriptional Activation by Estrogen Chromatin remodeling molecules Brg1, Brm and BAF155, and the histone acetylase (HAT) p300, are involved in estrogen receptor (ERα)-dependent transcriptional activation by estrogen (E2).

Brg1 has been reported to be involved in the activation of ERα-mediated transcription (Fig. 5) (DiRenzo et al. 2000). The potential involvement of prohibitin/Brg1/Brm in ER regulation was therefore investigated, and we found that prohibitin interacts with, and represses, ERα-mediated transcription after the binding of an estrogen antagonist to the ERα, through a mechanism requiring both Brg1 and Brm (Zhang et al. 2007). These findings indicate that the SWI/SNF complex participates in both the activation and the repression of ERα-mediated transcription, in a ligand-dependent fashion, and studies were next conducted to elucidate the mechanisms underlying the disparate functions of these chromatin remodeling molecules on the same promoters.

**Figure 6:**
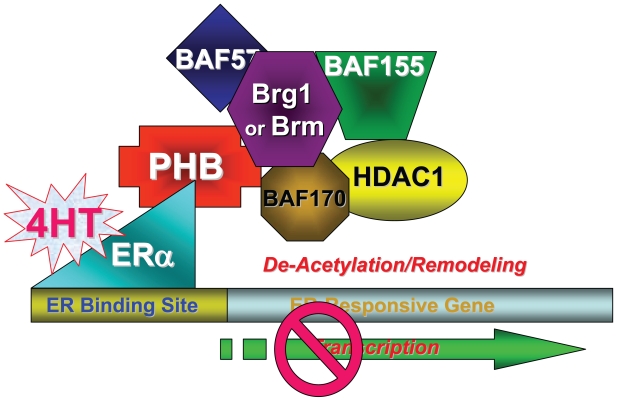
Repression of ER-Dependent Transcription by Estrogen Antagonists Members of the SWI/SNF complex (Brg1, Brm and BAF155, BAF170, BAF 57) and histone deacetylase 1 (HDAC1) are differentially recruited to ER-dependent promoters, *via* prohibitin (PHB) and estrogen receptor (ERα), for transcriptional repression in response to estrogen receptor antagonists such as tamoxifen (4HT).

The SWI/SNF complex contains protein components in addition to Brg1 or Brm, designated BAFs, based on their association with Brg1 (Battaglioli et al. 2002; Fryer and Archer, 1998; Huang et al. 2005; Kang et al. 2004; Lee et al. 1999; Letimier et al. 2007; Lickert et al. 2004; Rando et al. 2002; Takeuchi et al. 2007; Trotter and Archer, 2004; Underhill et al. 2000; Wang et al. 1996; Zhao et al. 1998; Zhao et al. 2005), raising the possibility that specific BAFs might participate in the differential regulation of ERα-mediated transcription by the SWI/SNF complex. Endogenous ChIP assays demonstrated that specific BAFs were differentially-recruited to the ERα-responsive promoters for activation versus repression. When SWI/SNF was recruited to ERα-responsive promoters directly by estrogen-bound ER, leading to activation, the complexes contained both BAF155 and BAF170. In contrast, when SWI/SNF was recruited to responsive promoters indirectly, through prohibitin recruited to an antagonist-bound ER, leading to repression, the complexes contained only BAF 155 (Fig. 6) (Zhang et al. 2007).

Further studies revealed that Brg1 and Brm participate in both transcriptional repression and activation on the same promoter, through ligand-specific differential collaboration with, and recruitment of, HDAC1 or p300, depending upon the composition of BAFs in the SWI/SNF complex (Zhang et al. 2007). siRNA knockdown studies demonstrated that BAF155 is necessary for the recruitment of both HDAC1 and p300 to responsive promoters, depending upon the ligand bound to the ER, whereas BAF170 is required only for p300 recruitment and ER-mediated transcriptional activation by estrogen, but not for HDAC1 recruitment and repression of ER-mediated transcription by estrogen antagonists. The recruitment of other additional members of BAF family to the ER-responsive promoters has also been detected, suggesting their potential involvement in ER-mediated transcriptional regulation. For example, BAF57 recruitment occurs in response to estrogen antagonists, but not to estrogen. The roles and mechanisms of the additional BAFs in ER-mediated transcriptional regulation remain to be elucidated (Fig. 6) (Zhang et al. 2007).

Prohibitin 2 is a protein closely-related to prohibitin, sharing a high degree of homology (Heron-Milhavet et al. 2007). A recent report that prohibitin 2 (known also as REA or BAP37) also associates with the estrogen receptor, and represses its activity, further demonstrates the importance of the prohibitin family in ER-mediated transcriptional regulation (Bitter, 2007; Delage-Mourroux et al. 2000; Gamble et al. 2007; Kurtev et al. 2004; Montano et al. 1999; Morrow et al. 2002; Murphy et al. 2000; Mussi et al. 2006; Park et al. 2005; Simon et al. 2000).

### Role of prohibitin in vitamin D-induced growth suppression of breast cancer

The active metabolite of vitamin D_3_, 1α,25(OH)_2_D_3_, plays an important role in regulation of cell proliferation and differentiation, in addition to its critical activity in bone mineralization, and analogs of vitamin D_3_ have demonstrated activity against breast cancers *in vitro* and *in vivo* (Hussain-Hakimjee et al. 2006; Peng et al. 2006; Peng and Mehta, 2007; Suh et al. 2004). Recent studies in breast cancer cells have identified prohibitin as the vitamin D target gene which participates in Vitamin D-induced growth suppression of breast cancer cells (Hussain-Hakimjee et al. 2006; Peng et al. 2006; Peng and Mehta, 2007; Suh et al. 2004). This discovery adds yet another aspect to the relevance of prohibitin to breast cancer treatment strategies.

## Prohibitin and Prostate Cancer

Prostate cancer is one of the leading causes of death and the most commonly diagnosed cancer in males (Bostwick and Meiers, 2007; Cummings, 2007; Freedland and Moul, 2007; Li, 2007; Lukka et al. 2006; Ryan and Beer, 2007). Prostate tumors depend, at least initially, upon circulating androgens for their growth and development (Aragon-Ching et al. 2007; Dehm and Tindall, 2007; Dudderidge et al. 2007; Emberton et al. 2007; Fleshner et al. 2007; Illing and Chapman, 2007; Lukka et al. 2006; Msaouel et al. 2007; Ryan and Beer, 2007; Shrivastava et al. 2007; Yee et al. 2007). A proteomic survey for androgen-responsive gene products identified prohibitin as a target gene of the androgen receptor, with the expression of prohibitin being transcriptionally repressed by androgens. Further studies suggested that prohibitin may play a role in the cellular growth response to androgen stimulation in prostate cancer cells and that prohibitin represses the androgen-dependent gene expression and growth of prostate cancer cells (Gamble et al. 2007; Gamble et al. 2004; Zhu et al. 2006).

The molecular mechanisms involved in the prohibitin-mediated regulation of the AR are incompletely understood (Gamble et al. 2007; Gamble et al. 2004; Zhu et al. 2006). A recent study indicates that prohibitin interacts with the androgen receptor indirectly. Over-expression of SRC1e, a co-activator for AR-dependent transcription, increased ligand-dependent AR activity, which was in turn suppressed by the over-expression of prohibitin in a dosage-dependent fashion, suggesting that prohibitin may compete against this co-activator in the modulation of AR activity. Furthermore, over-expression of prohibitin also caused a dissociation of ligand (androgen) from the AR (Gamble et al. 2007; Gamble et al. 2004; Zhu et al. 2006). Our very recent studies demonstrate that, analogous to the necessary role for prohibitin in the repression of ER-dependent transcription induced by estrogen antagonists, prohibitin is also required for repression of AR-dependent transcription induced by androgen antagonists (Yan, D. and Faller, D.V., unpublished data). Androgen antagonists induce recruitment of prohibitin to the AR complex on AR-responsive promoters, concomitant with recruitment of Brg1, but not Brm (in contrast to ER-dependent antagonist-induced repression, which requires both Brg1 and Brm). The precise mechanisms and clinical implications of prohibitin-mediated AR regulation are under active investigation.

The connection between prohibitin and prostate cancer was recently further illustrated by another study, which revealed that prohibitin participates in Transforming Growth Factor-β (TGF-β) signaling in prostate cancer cells (Zhu et al. 2006). TGF-β modulates growth, apoptosis, cell motility, and angiogenesis *via* a series of signal transduction events involving the phosphorylation of downstream receptors (Adler, 2007; Agarwal et al. 2006; Ao et al. 2007; Ding et al. 2006a; Ding et al. 2006b; Lauth and Toftgard, 2007; Narayanan, 2006; O’Connor et al. 2007; Rubenstein et al. 2006; Sampson et al. 2007; Sharifi et al. 2007; Shidaifat et al. 2007a; Shidaifat et al. 2007b; Tsai et al. 2007; Turley et al. 2007; Uchida et al. 2007; Wang et al. 2007; Yang et al. 2007; Ye et al. 2007a; Ye et al. 2007b). TGF-β acts as a tumor suppressor in prostatic tissues by inducing apoptosis and repressing cell proliferation in normal prostatic epithelium. Conversely, TGF-β promotes tumor progression and metastasis in tumor cells by inducing cell invasion, enhancing angiogenesis and immuno-suppression. To identify novel molecules involved in TGF-β signal transduction, a proteomic approach was employed and identified prohibitin as one of the proteins dramatically increased in response to TGF-β, suggesting that prohibitin may be a potential effector of TGF-β signaling. Subsequent investigation revealed that prohibitin was exported from nucleus in response to TGF-β. While the precise mechanisms and function of prohibitin in TGF-β-mediated prostate cancer apoptosis await further investigation, prohibitin appears to interact with Bcl2, an inducer of apoptosis, and this physical association between prohibitin and Bcl2 can be induced by TGF-β, suggesting the possible involvement of prohibi-tin/Bcl2 in the signaling events leading to apoptosis (Zhu et al. 2006). Depletion of prohibitin by siRNA suppressed TGF-β-mediated cell migration of prostate cancer cells, suggesting a potential role of prohibitin in the suppression of prostate tumor spread or metastasis.

## Additional Cancer-Relate Functions of Prohibitin

Ras signaling mediates the growth promoting signals initiated by many diverse growth factors and regulates normal cellular growth as well as malignant transformation. Earlier investigations have demonstrated that prohibitin interacts with Raf1, a central molecule of the MAPK pathway involved in Ras signaling (Wang et al. 1998; Wang et al. 1999b). A search for additional proteins involved in the regulation of apoptosis by a RNA-interference-based loss-of-function screen identified prohibitin as a required effector for Ras-mediated activation of MAPK and cell migration (Rajalingam et al. 2005). EGF receptors effect their growth promoting functions via Ras proteins, which in turn activate multiple diverging pathways involved in cell proliferation and differentiation, including the Raf-MEK-ERK cascade (Agarwal et al. 2006; Dhillon et al. 2007; Duh et al. 1997; El-Mowafy and White, 1999; Mandlekar and Kong, 2001; Margutti and Laufer, 2007; Rabenoelina et al. 2002; Rajalingam et al. 2005; Roberts and Der, 2007; Signorelli and Ghidoni, 2005). Depletion of prohibitin by siRNA blocked serum- or EGF-induced phosphorylation of the ERK and Raf1 signaling kinases, demonstrating a requirement of prohibitin in Ras-Raf1-MAPK signal transduction (Dhillon et al. 2007).

As mentioned previously, Raf1 interacts with, and regulates, prohibitin. The precise mechanism of prohibitin regulation by Raf1 remains undefined, but Raf1 kinase activity is required for this regulation. The reciprocal discovery of the necessary role for prohibitin in the activation of Raf1 and ERK suggests a possible feedback type of auto-regulation loop, in which prohibitin promotes Raf1 activation by Ras, leading to transduction of growth-stimulatory signals through the MAPK pathway, but Raf1 then also activates prohibitin, which suppresses the E2F node, thus providing a balance between growth suppression and proliferation. The newly-discovered roles of prohibitin in both transcriptional activation and growth suppression are consistent with such an auto-regulatory hypothesis, further delineating prohibitin as a critical modulator in the processes of transcriptional regulation, growth control and apoptosis.

In addition to the emerging importance of prohibitin in growth control in normal and transformed cells, the list of other functions ascribed to prohibitin is rapidly growing (Mishra et al. 2006; Mishra et al. 2005). As mentioned, at least a portion of prohibitin locates to the mitochondria, where it is believed that prohibitin chaperones imported proteins into the mitochondria (Wang et al. 2002a). An earlier report showed that prohibitin regulates the turnover of membrane proteins by the ATP-dependent protease of the inner membrane of mitochondria (m-AAA protease) (Steglich et al. 1999), and a recent study further linked prohibitin with the F(1)F(O)-ATP synthase complex, suggesting an alternative pathway for regulation of growth by prohibitin (Graef et al. 2007; Osman et al. 2007). The connections, if any, between these functions and the roles of prohibitin in growth control, apoptosis and signal transduction remain to be determined and reconciled. One potential link may stem from the discovery of the association between prohibitin and Bcl2, an anti-apoptotic protein located in mitochondria (Chiarugi et al. 1997). Elucidation of the role of mitochondrial prohibitin in apoptosis may lead to the identification of additional novel pathways connecting prohibitin with cancer development.

The recent and surprising finding of a potential connection between prohibitin and obesity elicited a response in the popular media (http://www.prohibitin.com/). Mishra, et al. attempted to develop a potential anti-obesity therapy based on targeted induction of apoptosis in the vasculature of adipose tissue, using *in vivo* phage display to isolate a peptide motif (sequence CKGGRAKDC) that homes to the vasculature of white fat. They identified that their fat tissue vasculature-targeted peptide bound to prohibitin, thereby establishing prohibitin as a marker of adipose vascular tissue (Mishra et al. 2005). Furthermore, targeting of a pro-apoptotic peptide to prohibitin in the adipose vasculature caused ablation of white fat, providing a potential basis for the design of a novel anti-obesity treatment. While any immediate connection between this finding and the roles of prohibitin in growth control is not apparent, it does illustrate the diversity of prohibitin’s functions. Furthermore, the concept of utilizing prohibitin and/or its related factors for inducing targeted-apoptosis might be exploitable as an anti-cancer strategy.

## Summary

Since the discovery of prohibitin as a protein with anti-proliferative activities more than a decade ago, major investigative efforts have resulted in the elucidation of the molecular mechanisms involved in prohibitin-mediated growth control. It is now clear that prohibitin is a transcriptional regulator, participating in both the activation and suppression of transcription. Further investigation into the clinical significance of this unique dual function may lead to better understanding of normal growth control and cancer development. The direct association between prohibitin and nuclear hormone receptor complexes (ER and AR) has further revealed the functions of prohibitin in transcriptional regulation of diverse genes and suggests the potential importance of prohibitin in breast and prostate cancers. Further translational studies to establish clinical importance of prohibitin signaling or loss in cancers may lead to the identification of novel strategies for the design of improved anti-cancer therapies. The requirement for prohibitin in one critical effector arm of Ras signaling also indicates the potential importance of prohibitin in signal transduction, and the possible existence of an auto-regulatory Raf1-prohibitin loop in the regulation of growth. Future investigation of the interplay between prohibitin and its diverse binding partners may lead to identification of novel molecular mechanisms regulating tumor cell and normal cell proliferation.

## Figures and Tables

**Table 1:** Binding partners of prohibitin.

	Proteins	Related functions	Citation
1	E2Fs	Transcription factor, cell cycle control	(Wang et al. 1999b)
2	Rb	Tumor suppressor	(Wang et al. 1999a)
3	P130	Tumor suppressor	(Wang et al. 1999a)
4	P107	Tumor suppressor	(Wang et al. 1999a)
5	P53	Transcription factor, tumor suppressor	(Fusaro et al. 2003)
6	IgM receptor	IgM signaling	(Terashima et al. 1994)
7	Raf1	MAPK signaling	(Wang et al. 1998)
8	Brg1	Chromatin remodeling	(Wang et al. 2002b; Wang et al. 2004)
9	Brm	Chromatin remodeling	(Wang et al. 2002b; Wang et al. 2004)
10	HDAC1	Chromatin remodeling	(Wang et al. 2002a)
11	HP1	Chromatin remodeling	(Rastogi et al. 2006a)
12	NCoR	Chromatin remodeling	(Wang et al. 2002a)
13	Estrogen receptor	Nuclear receptor	(Wang et al. 2004)
14	Androgen receptor	Nuclear receptor	(Gamble et al. 2007)
15	Bcl2	Apoptosis suppressor	(Chiarugi et al. 1997)
16	MLK2	JNK signaling	(Wang et al. 2002b)
17	SV40Tag	Viral-onco protein	(Wang et al. 2002b)

## Abbreviations

PHB: prohibitin
ChIP: chromatin immuno-precipitation
ER: estrogen receptor
BAF: Brg associated factors
